# Damage of *Streptococcus mutans *biofilms by carolacton, a secondary metabolite from the myxobacterium *Sorangium cellulosum*

**DOI:** 10.1186/1471-2180-10-199

**Published:** 2010-07-26

**Authors:** Brigitte Kunze, Michael Reck, Andreas Dötsch, André Lemme, Dietmar Schummer, Herbert Irschik, Heinrich Steinmetz, Irene Wagner-Döbler

**Affiliations:** 1 Group Microbial Communication, Helmholtz-Centre for Infection Research, Braunschweig, Germany; 2 Group Chronic Pseudomonas Infections, Helmholtz-Centre for Infection Research, Braunschweig, Germany; 3 Group Microbial Drugs, Helmholtz-Centre for Infection Research, Braunschweig, Germany; 4 Sanofi-Aventis Deutschland GmbH, Natural Products, Geb. H811, Brüningstr., 65929 Frankfurt, Germany

## Abstract

**Background:**

*Streptococcus mutans *is a major pathogen in human dental caries. One of its important virulence properties is the ability to form biofilms (dental plaque) on tooth surfaces. Eradication of such biofilms is extremely difficult. We therefore screened a library of secondary metabolites from myxobacteria for their ability to damage biofilms of *S. mutans*.

**Results:**

Here we show that carolacton, a secondary metabolite isolated from *Sorangium cellulosum*, has high antibacterial activity against biofilms of *S. mutans*. Planktonic growth of bacteria was only slightly impaired and no acute cytotoxicity against mouse fibroblasts could be observed. Carolacton caused death of *S. mutans *biofilm cells, elongation of cell chains, and changes in cell morphology. At a concentration of 10 nM carolacton, biofilm damage was already at 35% under anaerobic conditions. A knock-out mutant for *comD*, encoding a histidine kinase specific for the competence stimulating peptide (CSP), was slightly less sensitive to carolacton than the wildtype. Expression of the competence related alternate sigma factor ComX was strongly reduced by carolacton, as determined by a *pcomX *luciferase reporter strain.

**Conclusions:**

Carolacton possibly interferes with the density dependent signalling systems in *S. mutans *and may represent a novel approach for the prevention of dental caries.

## Background

Biofilms that harbour pathogenic bacteria are a serious health problem of increasing importance. They have been implicated in many persistent and chronic diseases such as cystic fibrosis, endocarditis, and infections caused by biofilms growing on incorporated foreign materials, e.g. stents, indwelling catheters, bone implants, and artificial valves [1-5]. Dental caries and periodontal diseases, which are among the most common bacterial infections in humans, are caused by biofilms known as dental plaque that result from microbial colonization of the tooth surface or the subgingival margin [6,7].

Eradication of biofilm bacteria by conventional antibiotic therapy is notoriously difficult or almost impossible due the much higher resistance level of the cells that is partially caused by the barrier effect of the exopolysaccharide matrix, and more importantly by profound genetic and metabolic adaptations of the cells to a sessile mode of growth [4,8,9]. It has been estimated that bacteria embedded in biofilms are more than 1000-fold less susceptible to the effects of commonly used antimicrobial compounds than are their planktonic counterparts [8,10,11]. Thus novel strategies for battling clinically relevant biofilms are urgently needed, particularly if one takes into consideration that biofilm-forming bacteria account for about two-thirds of human bacterial infections [10]. Quorum sensing systems might be promising targets in treating biofilm-induced infections. These intercellular communication mechanisms are mediated by extracellular small signalling molecules (autoinducers) and coordinate population wide gene expression of e.g. virulence factors such as biofilm formation in a cell-density-dependent manner [2,12]. The development of anti-quorum-sensing drugs that specifically attenuate virulence might represent an attractive alternative to conventional antibiotic therapy, particularly considering the fact that these antibacterial compounds are less likely to induce the development of resistant bacteria.

Such a "proof of concept" has already been provided by mimics of the acylated homoserine lactone signalling molecules, such as synthetic derivatives of natural furanones, which are able to inhibit *in vivo *biofilm development of *Pseudomonas aeruginosa *[13], and when covalently bound to surfaces also those of *Staphylococcus epidermidis *[14]. Moreover the quorum-sensing RNA III inhibiting peptide was shown to inhibit *in vivo *biofilm formation of *Staphylococcus aureus *[15]. However none of the quorum sensing blockers tested in animal models so far was suitable for human use.

Our project is aimed at finding new inhibitors of biofilm formation of the Gram-positive facultative anaerobic bacterium *S. mutans*. Among more than 500 bacterial species found in dental plaque [16-18]*S. mutans *is considered to be the principal pathogen causing human dental caries [19]. While metabolising dietary carbohydrates, *S. mutans *rapidly produces acid endproducts lowering the pH to approximately pH 3.5 resulting in demineralisation of the dental enamel and caries formation [20-22]. Moreover *S. mutans *can be a cause of subacute infective endocarditis [19,23].

We focused in our search for biofilm inhibitors on our collection of secondary metabolites from myxobacteria. They are ubiquitous Gram-negative soil bacteria characterized by their ability to glide in swarms, as well as by their complex life cycle that upon starvation culminates in the formation of multicellular fruiting bodies, containing dormant myxospores [24]. The complexity of their social behaviour and morphogenetic potential is reflected in their large genomes (9-13 Mbp), some of which, e.g. *Sorangium cellulosum *So ce56 [25] have been sequenced. The overrepresentation of genes involved in secondary metabolism also explains the capability of myxobacteria to produce such a high number of potentially useful low molecular weight compounds. Over the past 25 years, more than 100 secondary metabolites with more than 450 structural variants have been isolated from myxobacteria at the HZI. Most of these compounds turned out to be new and show novel unrelated structures as well as different biological activities with interesting mechanisms of action [24,26,27]. About a third of these myxobacterial compounds however did not show any biological or biochemical effect in our test battery, which until now predominantly focused at killing or inhibiting microbial growth. It should be pointed out that especially these compounds are of interest in exploring new targets.

**Figure 1:**
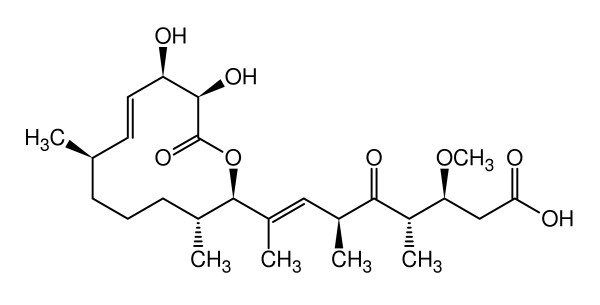
**Chemical structure of carolacton (from Ref. **[30]**, with permission)**.

Here we report on the efficacy of the secondary metabolite carolacton produced by *S. cellulosum *to biofilms of the cariogenic bacterium *S. mutans*. Figure 1 shows the structure of carolacton [28,29], the elucidation of which as well as its production and isolation have been reported elsewhere [30]. Carolacton induced damage of *S. mutans *biofilms at nanomolar concentrations, while planktonic growth was only weakly affected.

## Results

### Effect of carolacton on planktonic growth of bacteria and on eukaryotic cells

Carolacton has been reported to be inactive in standard bacterial growth inhibition tests using suspended (planktonic) cultures of Gram positive and Gram negative test strains [31] at least up to the highest tested concentration of 40 μg/mL (85 μM) [28]. The only sensitive strain was *E. coli *strain *tolC *(MIC 0.006 μg/ml) which is characterized by a defect in the TolC protein, a component of a multidrug efflux pump located in the outer membrane [32], making it hypersensitive to antibiotics. A minor antifungal activity (at 16 - 20 μg/mL) has been described against various filamentous fungi, e.g. *Aspergillus niger*, *Phytium debaryanum*, and *Sclertina sclerotiorum *[30]. Because of our biofilm screening results (see below) we determined the antibiotic activity of carolacton against *S. mutans *UA159 grown in planktonic culture. Carolacton only weakly inhibited growth under both aerobic and anaerobic conditions (MIC >106 μM) as determined in a conventional serial dilution assay. The turbidity of cultures (OD_620_) after 18-24 hours of incubation was reduced by 10-25% at concentrations of carolacton between 26.6 and 106 μM, respectively. Microscopical analysis showed that carolacton induced longer cell chains (see below), which might have contributed to the reduction in OD_620_.

Carolacton showed no acute toxicity in cell culture assays with L929 mouse fibroblasts. After 18 hours of incubation no inhibition of the metabolic activity of the cells was indicated by an MTT assay up to the highest tested concentration (79 μM). In all experiments the level of cytoplasmic histone-associated DNA fragments was below 1% of the positive control, thus no sign of apoptosis could be observed (again up to the highest tested concentration of 79 μM).

### Effect of carolacton on cell morphology and viability of *S. mutans*

**Figure 2:**
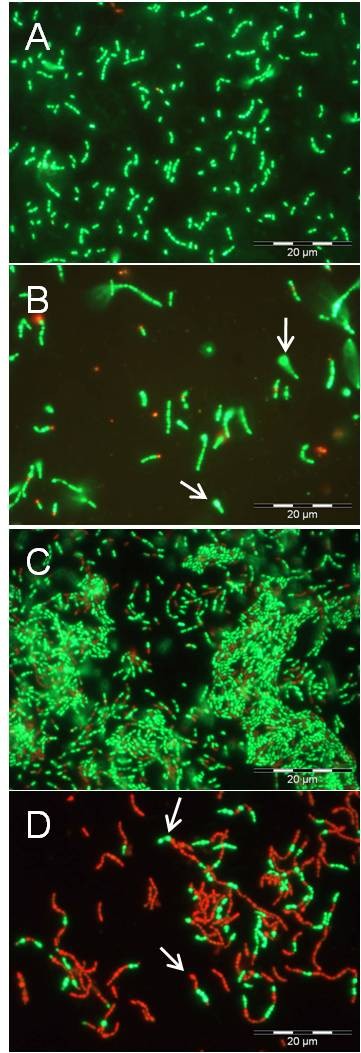
**Effect of carolacton on cell morphology and viability**. Fluorescent phase-contrast images of planktonically grown cultures (A, B) and biofilm cells of *S. mutans *(C, D) after LIVE/DEAD staining without (A, C) and in the presence of 5.3 μM carolacton (B, D). Planktonic cultures were grown in THB. Biofilms were grown in THB supplemented with 0.5% sucrose on microtitre plates for 24 h hours. Cultivation was at 37°C under anaerobic conditions (80% N_2_, 10% H_2_, 10% CO_2_). For microscopy, biofilm cells were scraped off from the bottom of the wells using pipette tips. Samples (100 μl) were stained with LIVE/DEAD BacLight bacterial viability staining kit L13152 (Molecular Probes; Eugene, OR, US) as recommended by the manufacturer and analysed using an Olympus BX60 microscope equipped with fluorescence filters U-MWB and U-MNUA2 and the Olympus digital camera Color View II (Olympus Optical Co., Ltd. Germany). Arrows (B, D) indicate bulging cells.

Phase contrast/fluorescence microscopy in combination with LIVE/DEAD *BacLight *bacterial viability staining (details see below) revealed that the majority of the biofilm cells of *S. mutans *grown anaerobically in the presence of carolacton (5.3 μM) showed red fluorescence, indicating damaged membranes and possibly death of the cells (Figure 2D), while planktonic cells were fluorescing green like untreated controls (Figure 2B). In addition, changes in cell morphology were observed, both in planktonic culture and in biofilms. In carolacton treated planktonic cultures cells appeared elongated, tended to form longer chains and some cells formed bulges, both as individuals and when growing in chains (Figure 2B), suggesting that cell division or acid tolerance could be influenced by carolacton. Nearly all of the planktonic cells were stained green, including also the balloon-like ones, which indicated that these cells too, were viable. Carolacton treated biofilm cells (Figure 2D) showed similar morphological modifications, yet many of the cells, including also most of the balloon-like ones, were stained red. Thus, the membrane damage resulting from carolacton treatment appears to be specific for biofilm cells. Although the microscopical observations in Figure 2 are not quantitative, they confirm that carolacton treated planktonic cultures had a slightly reduced density compared to untreated controls.

### Quantification of *S. mutans *biofilm damage by carolacton

**Figure 3:**
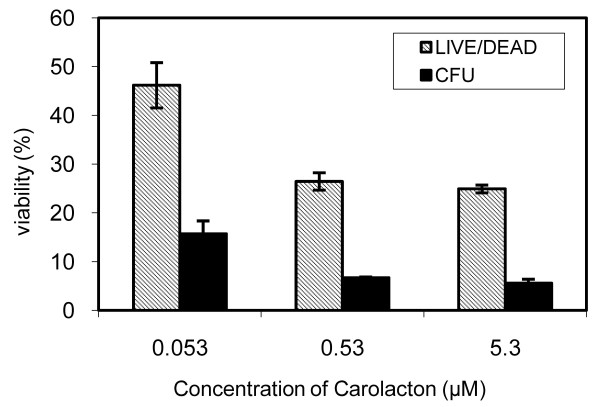
**Quantification of the viability of carolacton treated *S. mutans *biofilms determined by counting colony forming units (CFU) and by measuring membrane damage, calculated as the green/red ratio after LIVE/DEAD BacLight Bacterial Viability staining in percent of untreated controls**. Biofilms were grown for 24 h under anerobic conditions. Each data point is the average +/- standard deviation of triplicate to fourfold determinations. The CFU in the control without carolacton was 2.1 × 10^7^ml^-1^.

We attempted to quantify the extent of biofilm damage caused by carolacton by determining colony forming units (CFU). Figure 3 shows that the number of CFU in carolacton treated biofilms was only 5 - 15% compared to untreated controls, thus confirming that carolacton induced cell death. Due to the microscopic observations described above, these results have to be interpreted cautiously, because not only the high percentage of red stained biofilm cells, but also the elongated cell chains reduced the viable cell count. Disaggregation of these chains by sonification failed to yield individual cells or short chains comparable to untreated cultures and led to more or less complete cell death.

Therefore, we used the LIVE/DEAD *BacLight *bacterial viability staining as a sensitive and fast method for quantifying the effect of carolacton on biofilm viability of *S. mutans*. Biofilm damage was calculated as the ratio of green versus red fluorescence of the biofilm cells normalized against the untreated control. This parameter is indicative of the membrane integrity of the cells, since the red fluorescing dye can only enter cells with damaged membranes. For *S. mutans*, a calibration curve using isopropyl alcohol-killed and live cells in varying proportions resulted in a linear correlation between the ratio of green to red fluorescence and the amount of live cells (data not shown). For carolacton treated cells, Figure 3 shows that the extent of biofilm damage calculated from fluorescence staining was much smaller than that obtained by CFU counting. Thus, the green/red ratio of fluorescence is a conservative estimate of biofilm damage in *S. mutans*.

### Dose-dependent damage of biofilms of *S. mutans *by carolacton

**Figure 4:**
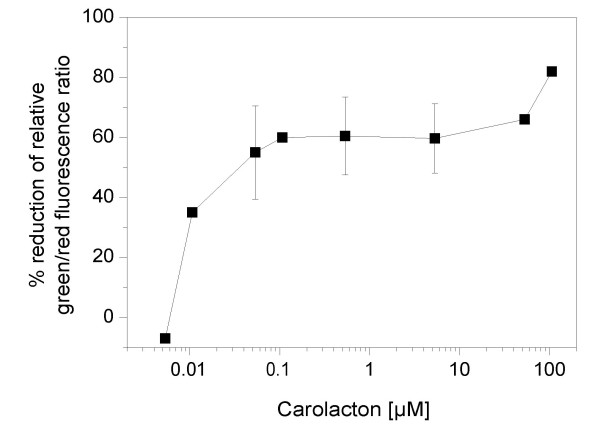
**Effect of carolacton concentration on the membrane damage of *S. mutans *biofilms**. Biofilms were grown for 24 h under anaerobic conditions and stained using the LIVE/DEAD BacLight Bacterial Viability kit. Green and red fluorescence was determined, and biofilm damage was calculated as reduction of the fluorescence ratio green/red compared to untreated controls. Each data point is the average of triplicate samples. Standard deviations are given for data points determined in at least three independent experiments.

Biofilm damage was determined for 24 h old biofilms of *S. mutans *grown under anaerobic conditions, which predominate in dental plaque, using concentrations of carolacton between 0.0053 μM and 106.5 μM. As shown in Figure 4, carolacton decreased biofilm viability over a concentration range of three orders of magnitude (from 0.053 μM to 53 μM) to approximately the same degree (55 - 65%). At a concentration of 0.01 μM (5 ng/ml) carolacton, biofilm damage was already 35%. This type of dose-response relationship is typical for quorum sensing controlled processes. A very low inducing threshold concentration is followed by a broad saturation range, resulting in the lack of a linear relationship between signal concentration and response [33].

### Time course of biofilm damage by carolacton

**Figure 5:**
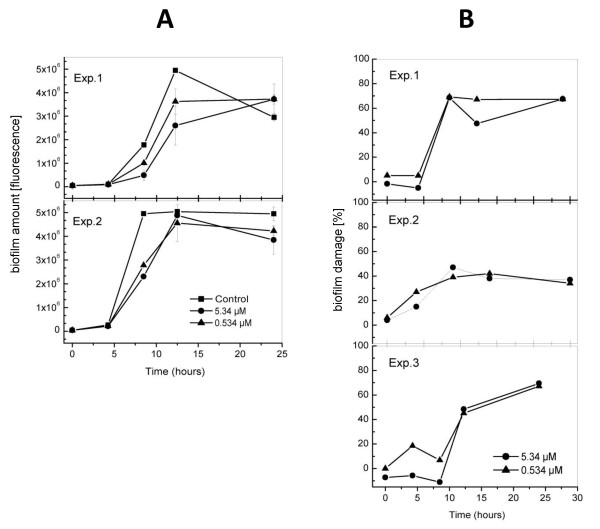
**Time course of biofilm growth of *S. mutans *in the presence and absence of carolacton**. A Biofilm volume, determined by using only the SYTO9 (green) component of the LIVE/DEAD BacLight Bacterial Viability stain. Two representative experiments are shown. Green fluorescence, which is a measure of total biomass, is shown in absolute units. B Biofilm membrane damage, determined using the LIVE/DEAD BacLight Bacterial Viability stain. Green and red fluorescence was measured, and biofilm damage was calculated as reduction of the ratio of green/red fluorescence compared to controls without carolacton. Error values were calculated from the standard deviations of the green/red ratios of control and carolacton treated samples according to the error propagation formula of Gauss. Three representative experiments are shown. Biofilms were grown anaerobically. Mean and standard deviation are given for triplicate samples.

We next investigated the dynamics of biofilm growth and its disturbance by carolacton during the first 24 h under anaerobic conditions. Green fluorescence of biofilms stained with SYTO9 alone is a measure of the total amount of biofilm cells, both alive and membrane damaged, and was applied here to study the growth of *S. mutans *biofilms with and without carolacton. Figure 5A shows two typical time courses for biofilm growth. In the untreated control, the amount of biofilm cells reached its maximum after 8 - 12 h, followed by a plateau and sometimes by a slow decrease, presumably due to detachment of biofilm fragments in the mature biofilm. During these first 12 h of biofilm growth, carolacton (5.3 μM) reduced the total amount of biofilm cells, as determined by total green fluorescence, up to 54%, but this effect was not observed any more after 24 h.

Membrane damage of the biofilm cells, determined by the LIVE/DEAD *BacLight *fluorescence staining method by staining with both SYTO9 (green) and propidium iodide (red), was calculated as the reduction of the green/red fluorescence ratio in biofilms grown with carolacton relative to untreated controls and is shown in Figure 5B for three independent experiments. It shows a similar pattern. Biofilm damage was small during the first 6 h, increased rapidly until about 8.5 or 12.25 h, respectively and then remained stable or increased more slowly till the end of the experiment after 24 hours. The curves for the two concentrations of carolacton tested were very similar, as expected from the concentration range of carolacton activity determined previously (Figure 4). The maximum reduction of the relative green/red fluorescence ratio was between 47% and 69% reflecting the dynamic process of biofilm growth. The pH dropped from pH 7.8 to pH 4.3 (24 h of growth), but there was no difference in controls and carolacton treated cultures. To summarize, the data show that carolacton temporarily reduced the total amount of biofilm cells, indicated by staining with the green fluorescent dye alone, during the period of maximum biofilm growth (Figure 5A). Most importantly, carolacton strongly reduced the viability of cells within the biofilm, determined by the reduction of the relative proportion of green to red fluorescence, throughout 24 h of biofilm development but mainly during the period of maximum biofilm growth and thereafter, while little reduction of viability was observed during the initial hours of biofilm growth (Figure 5B).

### Investigation of the effect of carolacton on *S. mutans *biofilms by confocal laser scanning microscopy

**Figure 6:**
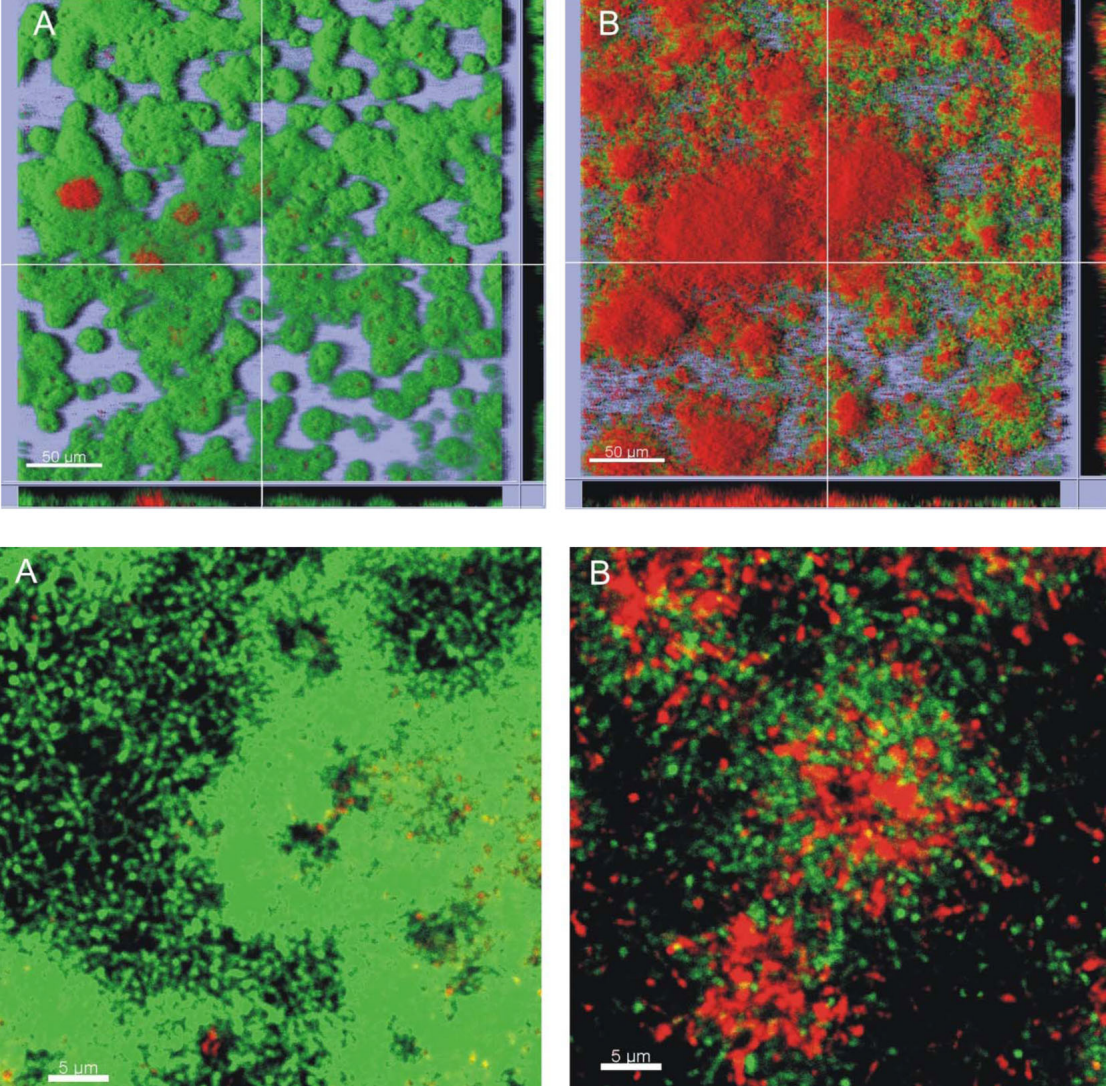
**Confocal laser scanning microscope images of *S. mutans *biofilms in the absence (A) or presence (B) of 0.5 μM carolacton after 12 h of anaerobic cultivation**. Staining using the LIVE/DEAD BacLight Bacterial Viability Kit assessed bacterial viability: green areas indicate live cells; red areas indicate dead or damaged cells. The top panel shows a bird's eye view on the biofilm with lines indicating the position of the vertical sections shown at the lower and right margins of both images. Acquired using an UPLSAPO 20× objective lens, size of scale bar 50 μm. The bottom panel shows enlarged horizontal sections of *S. mutans *biofilms in the absence (A) or presence (B) of 0.5 μM carolacton, aacquired using an UPLSAPO 40× objective lens with 7× digital magnification, size of scale bar 5 μm.

The effect of carolacton on the spatial distribution, architecture and viability of biofilms of wild-type *S. mutans *UA159 was investigated by confocal laser scanning microscopy. Figure 6A shows top-down views, flanked by pictures of vertical optical sections after 12 hours of cultivation and Figure 6B represents horizontal sections at a higher magnification. Control biofilms showed a characteristic architecture with pancake like cell clusters distributed randomly over the substratum, occasionally growing together but leaving biofilm free spaces in between them. Control biofilms also showed rare signs of membrane damage which initiated at the substratum-oriented side of the biofilm. In biofilms grown in the presence of carolacton, a significant part of the cells was stained red, indicating that cell membrane integrity was severely damaged. Vertical optical sections show that membrane damage occurred throughout the biofilm, at the substratum-oriented side as well as towards the biofilm surface. Biofilm architecture appeared less dense than in the controls, and small cell clusters were scattered across the substratum with little empty space in between them. The magnification of the biofilms (Figure 6B) shows that the central regions of cell clusters were affected most by carolacton.

### Effect of carolacton on biofilms of quorum sensing negative mutants

*S. mutans *utilizes a density-dependent quorum sensing signalling system to regulate the expression of virulence factors, including biofilm formation. It involves an excreted autoinducer, the competence stimulating peptide (CSP) encoded by *comC*, which is detected by a two-component signal transduction system comprising the histidine kinase ComD and the response regulator ComE [34-38].

**Figure 7:**
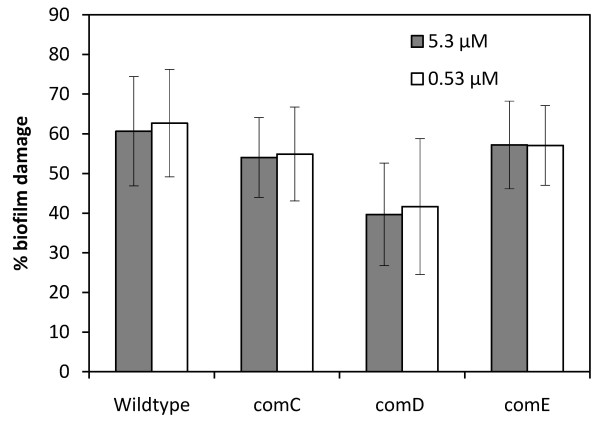
**Damage of biofilms of *S. mutans *wildtype and knock-out mutants for *comC*, *comD *and *comE *by carolacton**. Biofilms were grown under anaerobic conditions for 24 h and stained with the LIVE/DEAD BacLight Bacterial Viability staining kit. Green and red fluorescence was determined in triplicate samples, and biofilm damage was calculated as reduction of the fluorescence ratio green/red compared to untreated controls. Standard deviations were calculated from 3 - 5 independent experiments.

To find out if carolacton interferes with this system, we tested its effect on biofilm formation of knockout mutants for *comC*, *comD *and *comE*. Biofilms were grown under anaerobic conditions in the presence of 0.53 μM or 5.3 μM carolacton, respectively, and stained and analysed as described after 24 h of biofilm growth. For each strain and carolacton concentration, between 3 and 5 experiments were carried out. The green/red fluorescence ratio for untreated controls was the same for the wildtype and the three mutants. Figure 7 shows that biofilms of the wild-type strain *S. mutans *were damaged by carolacton with an average level of 61% (5.3 μM carolacton) or 63% (0.0.53 μM carolacton). *comC *and *comE *mutants showed slightly lower mean inhibition values, but this difference was not statistically significant. Biofilms of the *comD *mutant were only damaged by 40% (5.3 μM carolacton) or 42% (0.53 μM carolacton) and the difference to the wildtype was statistically significant for both tested concentrations (one sided Student's t-test, p = 0.019 and p = 0.032, respectively).

Thus, the *comD *knockout mutant was slightly less sensitive to carolacton than the wildtype. This could indicate that carolacton interferes with the membrane bound histidine kinase ComD. However, since the *comC *and *comE *mutants were just as sensitive for carolacton as the wildtype, and since there was still considerable activity of carolacton against the *comD *mutant, other mechanisms must be more important.

### Influence of carolacton on a *pcomX *luciferase reporter strain

**Figure 8:**
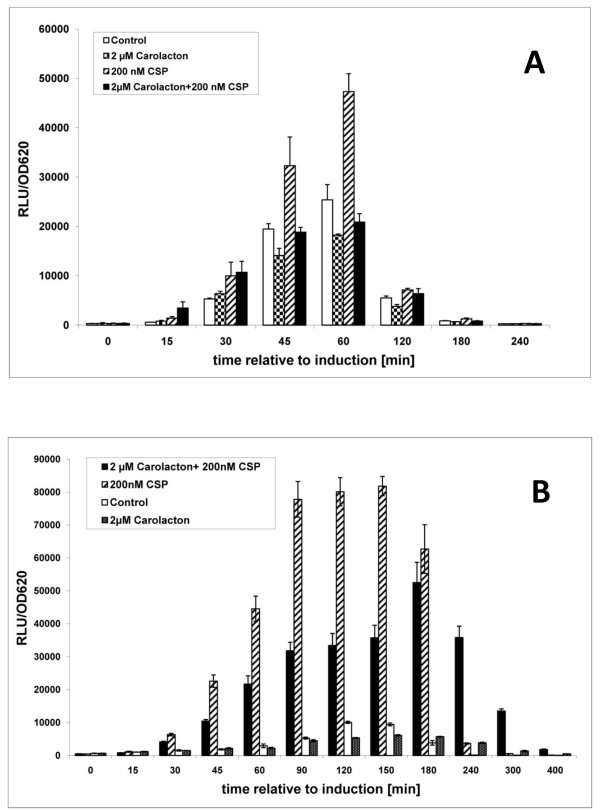
**Effect of carolacton on the *comX*-promoter activity of *S. mutans***. The CSP-induced *comX-luc *gene expression in the reporter strain construct was determined in the presence and absence of 2 μM carolacton. The luciferase activity was normalized against the optical density at 620 nm and measured for different time-points after induction of luciferase expression with 0.2 μM CSP. The expression of *comX-luc *in cultures which were not induced by externally added CSP and its inhibition by carolacton is also shown. Cultures were grown under anaerobic conditions as biofilms (A) or in suspension (B).

ComX, an alternative sigma-factor, plays a key role in the quorum sensing system of *S. mutans *which controls not only genetic competence, but also stress tolerance and biofilm formation, leading to the suggestion to call it the "X-state" rather than competence [39]. ComX is positively induced by CSP through the response regulator ComE, but also by another two component system, CiaRH, and environmental stress [40]. ComX controls the late competence genes, including the machinery for DNA-uptake and processing, but also many other density dependent traits [36,40-42]. Altogether 240 genes are directly or indirectly controlled by ComX [42]. To investigate the effect of carolacton on the promoter activity of *comX *a *pcomX*-luciferase reporter strain was constructed. For the experiment a concentration of CSP (200 nM) was chosen that induced competence without causing substantial growth inhibition [42]. Figure 8A shows that a severe reduction of CSP-induced *comX *expression was caused by addition of carolacton to biofilms grown anaerobically. Furthermore carolacton led to a decrease of growth-dependent, basal *comX*-reporter activity. Maximum inhibition was seen 60 min post induction at the peak of *comX *expression. In planktonic culture (Figure 8B) similar results were obtained, but both the CSP induced expression of *comX *and its inhibition through carolacton occurred over a longer time, e.g. from 45 to 180 min post induction, possibly reflecting the lower cell density in the planktonic culture. Furthermore we found that carolacton reduced the growth-dependent *comX*-promoter activity of this reporter strain also in the absence of externally added CSP, both in biofilms and in planktonic culture.

## Discussion

Dental caries, gingivitis, and periodontal diseases, which may develop as a consequence of dental plaque formation, are among the most common bacterial infections in humans. Eradication of cariogenic bacteria within dental plaque is notoriously difficult and therefore new drugs and drug applications are constantly being tested. In this study we successfully explored the possibility to use secondary metabolites from a group of soil bacteria producing diverse novel structures with a large spectrum of mechanisms of action, as inhibitors of biofilms of *S. mutans*, a bacterium which plays a key role in dental biofilm formation and dental caries. One such compound, carolacton, proved to strongly disturb biofilm formation of *S. mutans*.

Carolacton has been isolated from a myxobacterium of the species *S. cellulosum*, and was among the substances which were not developed further because it was "inactive", e.g. showed no significant antibiotic or antifungal activity nor acute cytotoxicity. The new biofilm screen described here resulted in the discovery of a promising biological activity for carolacton. Our study clearly demonstrates that carolacton showed high antimicrobial activity against biofilms of *S. mutans*, while planktonic growth of bacteria, including *S. mutans*, was only slightly affected. Thus, carolacton appears to target a mechanism specific for biofilm development of *S. mutans*.

The data show that in biofilms carolacton causes membrane damage and cell death as well as morphological changes, e.g. elongated cells, increased chain length and bulging. Total biofilm mass was only temporarily reduced during the first 12 h of biofilm growth, but not in the later stages under the conditions tested here. The dose-response curve of the activity of carolacton showed a very low threshold concentration of 10 nM and no substantial increase of activity above this concentration, suggesting that it acts as a trigger/inhibitor of a signalling pathway. We hypothesized that carolacton might induce cell death and possibly reduced acid tolerance (resulting in elongated or bulged cells) by interfering with the competence and stress related cell-cell signalling network in *S. mutans*. This network is comprised in part of pheromone CSP (the *comCDE *system)-dependent and CSP independent components which respond to environmental signals [40,42,43]. CSP can trigger cell death at high concentrations by inducing an auto-active intracellular bacteriocin, CipB, in a fraction of the biofilm cells [42]. In some ways the activity of carolacton is similar to that of CSP at concentrations above those that induce competence. CSP and carolacton both induce balloon like cell morphology, and cell death in about 50% of the biofilm cells, an effect which was not increased by increasing their concentration [33]. Unlike carolacton (see below), CSP activity is exclusively mediated through *comDE*, i.e. the *comC *and *comD *null mutants were insensitive to CSP [33]. We studied the response of mutants lacking functional *comC*, *comD *or *comE *to carolacton. Only the *comD *mutant showed slightly less biofilm damage than the wildtype. The histidine kinase ComD induces transcription of the "early" competence genes, among them 5 mutacins and the sigma factor ComX. ComX then triggers the expression of the "late" competence genes. The lack of ComD controlled synthesis of mutacins, among them an autolysin, and their corresponding immunity proteins and membrane transporters, and the reduced expression of the late competence genes, including stress tolerance genes, in the Δ*comD *mutant strain, apparently makes this mutant more resistant to carolacton, although only to a small extent.

However, other mechanisms must be operating as well, since this mutant was still damaged by about 40%. Fourteen two-component systems consisting of a histidine kinase (HK) and a response regulator (RR) have been identified in *S. mutans *[44,45]. In addition to ComDE, genetic competence is also mediated through VicRK (HK/RR1) [46], the CiaHR (HK/RR2) [40], and the HK/RR11 [36,47]. Moreover, immunity against autolysis is controlled in a density dependent way by LiaSR (formerly HK/RR11)[48]. Carolacton might therefore act not only or not primarily on ComD, but also on some of the other two component systems of *S. mutans*.

To obtain further insights into the possible mode of action of carolacton, we then studied its effect on the expression of ComX, the alternate sigma factor of *S. mutans *which is induced by CSP and stress and controls not only genetic competence [41], but also stress related traits. Altogether 240 genes are directly or indirectly controlled by *comX *[42]. The data show that indeed the expression of *pcomX *after induction by CSP is strongly inhibited by carolacton, suggesting that carolacton interferes with the ComX related signalling network in *S. mutans*. The alternate sigma factor ComX controls the expression of the so-called "late" competence genes. They comprise the complete cellular machinery for uptake and processing of DNA, representing the essential mechanism for genetic competence. In addition, stress related phenotypes are also controlled by *comX *[42]. Competence is not only induced by the ComDE mediated signaling cascade, but several other two-component systems and response regulators are also involved, e.g. CiaH, HtrA [40], HK11/RR11 [47], and the VicRK system [46]. Therefore, CSP related and CSP independent signaling cascades exist for inducing competence and stress tolerance, which however all rely on the late competence genes and thus converge on *comX *induction. A reduction in the expression of *comX *by carolacton after CSP stimulation could therefore be caused by a direct interaction of carolacton with ComD, with CSP, or with the binding of CSP to ComD, resulting in an impaired signaling cascade and reduced *comX *expression. Since a Δ*comD *mutant, which cannot respond to CSP through ComD, shows only slightly reduced sensitivity to carolacton, this scenario is not supported by the data. It appears more likely that one of the other two-component systems involved in competence regulation or stress response is inhibited by carolacton, and that this inhibition is relayed to *comX *via the specific signaling cascade. The *comD *gene was shown to be differentially expressed in a RR11 mutant [47], and therefore an indirect effect of carolacton on *comD *expression through one of the other two-component systems is also possible.

Other mechanisms could also contribute to cell death in a growth dependent way. For example, the gene *atlA *was discovered to decrease autolysis and cause elongated cell chains, thus affecting biofilm formation [49,50]. Interestingly, the Δ*comD *mutant, which is unable to induce *comX *expression after CSP stimulation, was slightly less sensitive to carolacton, but carolacton reduced the CSP induced *comX *expression, which appears to be contradictory. However, the sigma factor *comX *and the histidine kinase *comD *are connected through a complex signaling network which receives input from several histidine kinases as well as additional regulators. The experimental conditions analysed here, e.g. knock-out of *comD*, and determination of *comX *expression after CSP stimulation, both are highly artificial. Thus, since the mechanism of carolacton is not known, the causal relationship between them cannot be inferred from the data presented here. ComD plays apparently only a small role for the effect of carolacton. If one or several of the other thirteen two-component systems of *S. mutans *are affected by carolacton, this could lead to the observed result with the highly sensitive *pcomX *reporter strain. A transcriptome analysis would be needed to determine the effect of carolacton on *comD *and *comX *expression as well as on the other two-component systems of *S. mutans *under "natural" conditions, d.h. without additional stimulation by CSP.

CSP has been shown to inhibit biofilms and to cause elongated cells at high concentrations [33]. Antibacterial activity of other peptides has been tested against *S. mutans*, but relatively high concentrations are required [51]. Killing activity was therefore enhanced by a combination of inhibitory peptides with desinfectants [52]. Killing activity has also been enhanced by constructing synthetic peptides consisting of two inhibitory domains [53]. In another approach, the cytotoxic effect of inhibitory peptides was combined with the specificity of the ComD receptor, resulting in so called STAMPs (targeted antimicrobial peptides). Such STAMPs were constructed by fusing an inhibitory peptide with a targeting domain derived from CSP [54]. By contrast, carolacton is structurally unrelated to peptide pheromones. Proof of principle for using chemically unrelated compounds as inhibitors has been obtained for the acylated homoserine lactone based quorum sensing system of Gram negative bacteria [55].

## Conclusions

Bacterial signalling systems have emerged in recent years as attractive targets for antimicrobial therapy. The discovery of a compound damaging *S. mutans *biofilms which might be targeting one or several of its two-component systems involved in regulating biofilm formation, autolysis and stress tolerance could provide a novel approach for future therapeutic strategies to prevent dental plaque related diseases with only minimal impact on the normal microbial flora.

## Methods

### Bacterial strains and culture conditions

*S. mutans *wild-type strain UA159 (ATCC 700610) and its knockout mutants defective in the quorum sensing genes *comC, comD*, or *comE *have been provided by courtesy of Prof. Dr. D. G. Cvitkovitch from the University of Toronto, Canada. The mutants were constructed by allelic replacement of the gene in question with an erythromycin resistance cassette using the PCR ligation mutagenesis strategy described in more detail in [56]. The wild-type strain was maintained routinely on Todd-Hewitt (TH) agar plates (Difco) and liquid cultures were grown in Todd-Hewitt broth Bacto™(THB). For cultivation of the mutants, erythromycin was added at 10 μg per ml to the media. For biofilm growth, THB was supplemented with 0.5% sucrose (THBS). Incubation was at 37°C without agitation under aerobic (with 10% CO_2_) or anaerobic (80% N_2_, 10% H_2_, 10% CO_2_) conditions. For anaerobic growth, the medium was flushed with nitrogen before use. *Escherichia coli *DH5α was used as cloning strain and routinely cultured in Luria Bertani (LB, Carl-Roth, Karlsruhe, Germany) medium at 37°C. *E. coli *strains carrying plasmids were selected with 50 μg ml^-1 ^spectinomycin.

### Inhibition of planktonic growth and determination of cytotoxicity

The minimal inhibitory concentration of carolacton on planktonic growth of *S. mutans *UA159 was determined with the conventional serial two-fold dilution method in 96-well microtiter plates (200 μl/well). As inoculum 1 × 10^6 ^cells/ml were used, and carolacton was dissolved in MeOH, producing concentrations in the cultures of not more than 5%. Incubation was for 24 hours at 37°C under both anaerobic and aerobic conditions. Optical density (OD) measurements at 620 nm were performed using a Wallac Victor3™1420 Multilabel Counter (Perkin-Elmer Life Sciences). Acute cytotoxicity against L929 mouse cells (connective tissue, ATCC CCL 1) was determined using an MTT assay as reported [57]. Cytoplasmic histone-associatd DNA fragments were measured with the Cell Death Detection ELISA kit from Roche Diagnostic to determine apoptosis induction in L929 cells. Cells were inoculated at 10000 cells/well in a 96-well plate, grown to sub-confluence for 2 days and then incubated with different concentrations of carolacton for 18 hours.

### Biofilm cultivation

Biofilm formation was induced in 96-well polystyrene flat-bottom microtiter plates (Greiner bio-one, μClear-Plate Black). Overnight cultures of *S. mutans *UA159 and its corresponding mutants grown anaerobically in THB (if necessary in the presence of 10 μg/ml erythromycin) were diluted to an OD_620 _of 0.01-0.03 in fresh THB with the addition of 0.5% (w/v) sucrose. Aliquots thereof (95 μl) were distributed into microtiter plate wells, which contained 5 μl of different concentrations of a test compound or alternatively 5 μl of methanol as control. All measurements were done in triplicate. The microtiter plates were incubated at 37°C without shaking under anaerobic conditions for 24 h unless indicated otherwise.

### Determination of cell viability by counting colony forming units (CFU)

Samples were serially diluted in 0.85% NaCl, and two to three appropriate dilutions were plated in triplicate onto TH agar and incubated anaerobically at 37°C for 2 days before counting. For enumerating biofilm CFUs, biofilms were scraped off from the bottom of the wells using pipette tips, resuspended in 0.85% NaCl, vortexed for 1 min and treated as above.

### LIVE/DEAD *Bac*Light bacterial viability staining

Biofilms were analysed using the LIVE/DEAD *Bac*Light bacterial viability staining kit L13152 (Invitrogen, Molecular Probes, Inc. Eugene, OR, USA) according to the manufacturer's instructions. The kit consists of two stains, propidium iodide and SYTO9, which both stain nucleic acids. When used alone, green fluorescing SYTO9 generally labels all bacteria in a population, whereas red fluorescing propidium iodide only penetrates bacteria with damaged membranes, causing a reduction in the SYTO9 stain fluorescence. Thus with an appropriate mixture of the SYTO9 and Ppropidium iodide stains, bacteria with intact membranes stain fluorescent green, and bacteria with damaged membranes stain fluorescent red. Staining of biofilms was usually carried out for 15 min in the dark at room temperature with 100 μl of a 1:1 mixture of the two dye components. In some experiments biofilms were also stained exclusively with the green fluorescing component SYTO9. To remove planktonic and loosely bound bacteria the biofilms were carefully washed before staining with 100 μl of 0.85% NaCl. Fluorescence was measured in a microtiter plate reader (Wallac Victor3™1420 Multilabel Counter, Perkin-Elmer Life Sciences) equipped with detectors and filter sets for monitoring red (630 nm) and green (535 nm) fluorescence. Results are expressed as reduction of the ratio of green/red fluorescence compared to untreated controls.

### Construction of a *pcomX *luciferase reporter strain and luciferase assay

For the construction of the luciferase reporter strains, the advanced firefly luciferase gene was amplified using *Pfu *polymerase from plasmid pHL222 (Lößner *et al. *, unpuplished) by utilisation of primers lucF (5'ATATA**CCATGG**AAGACGCCAAAAAC) and lucR (5'-AAAAAA**ACTAGT**TTATGCTAGTTATTGCTCAGCGG-3') bearing restriction sites (bold) for NcoI and SpeI. The amplicon was cloned into the suicide vector pFW5 [58] via the NcoI and SpeI sites to generate plasmid pALEC15. A fragment comprising approximately 1 kb of sequence upstream of the *comX *start codon was PCR-amplified using genomic DNA of *S. mutans *UA159 as template

(Primer pair P102_1997 For (5'-AAAAAAA**CCATGG**TCCAAAAATAAGTGACTAAGG-3') and P103_1997 Rev (5'-AAAAAAA**CCATGG**CTATTACGATGACCTCCTTT-3')). Restriction sites for NcoI (bold) were introduced via the 5' termini of the PCR primers. The digested amplicon was ligated into the vector pALEC15 cut with the same enzyme and containing the promoterless luciferase gene and a spectinomycin resistance cassette. Constructs confirmed by PCR and sequencing were transformed in *S. mutans *UA159 according to the method of Li *et al *[34] and chromosomally integrated via single crossover homologous recombination. Transformed cells were plated on selective THY agar with spectinomycin (600 μg/ml) and single colonies were picked. For the confirmation of the expected integration a PCR was performed and the identity of the integrated DNA was confirmed by sequencing In addition the inductivity of clones with CSP was tested as positive control [41]. The luciferase assay was performed in optical 96 well polystyrene white microtiter plates (Nunc) as described by Loimaranta *et al. *[59]. Briefly, overnight cultures of the *pcomX*-luciferase reporter strain of *S. mutans *were diluted 1:10 in fresh THB-media (pH 6.5) and grown for one hour at 37°C under anaerobic conditions. Aliquots of 100 μl of cells were taken as reference sample before CSP-induction. Subsequently 2 μM carolacton and/or 200 nM CSP were added to the cells and samples were taken at different timepoints post induction. The production of luciferase was stopped by an immediate cold-shock and an incubation on ice. In addition the luminescence of untreated cells was also determined. For the assay 100 μl of the samples were diluted with 100 μl of glucose-containing buffer (2% glucose, 0.9 mM ATP, 25 mM tricine, 5 mM MgSO_4_, 0.5 mM EDTA, 0.5 mM DTT to ensure sufficient levels of intracellular ATP. After incubation for 10 minutes at room temperature 100 μl of 360 μM D-luciferin in 20 mM tricine was added through a dispenser and luminescence was measured in a Victor X-Light™1420 Luminescence Plate Reader (Perkin Elmer Life Sciences). For an appropriate comparison of the different samples the luminescence was normalized against the optical density at 620 nm wavelength. The mean of at least three independent biological samples was determined, and each experiment was repeated at least twice.

For the determination of *pcomX *controlled luciferase activity in biofilms, an overnight culture of the *S. mutans pcomX*-luciferase reporter strain was diluted in fresh THBS-medium to an OD_600 _= 0,05. The biofilms were grown in an polystyrene 96 well microtiter-plate (Greiner BioOne, Frickenhausen, Germany) containing 200 μl of cell culture per well. After 3,5 h of growth (37°C, anaerobic conditions) the supernatant was completely removed and replaced with fresh THBS-medium containing 200 nM CSP and/or 2 μM carolacton. Untreated cells were used as reference samples. At least three wells were used as replicates for each condition tested. Samples were harvested at different time points following supplementation of CSP and/or carolacton using a rubber scraper. Scraped off cells were resuspended in 200 μl of THBS and the luciferase assay was performed as described above.

### Confocal Laser Scanning Microscopy

Biofilms developed on half area 96-well polystyrene flat-bottom microtiter plates for 12 or 23 h in triplicate and stained with the LIVE/DEAD BacLight viability kit (see above) were observed using an Olympus FlowView 1000 (Olympus, Tokyo, Japan) confocal laser scanning microscope. To acquire green ("live") and red ("dead") fluorescence, respectively, a laser excitation at 488 nm (Ar laser) and 561 nm (He laser) and emission filters at 500 - 545 nm and 580 - 680 nm were used. Image data were subsequently processed with the Imaris software (Bitplane AG, Zürich, Switzerland).

## Authors' contributions

BK conducted the biofilm screening experiments, characterized carolacton activity, and, together with AD, did the confocal laser scanning microscopy. MR and AL constructed the *pcomX *reporter strain and determined *pcomX *activity. DS, HI and HS discovered, isolated and purified carolacton from bacterial cultures. IWD drafted the study and together with BK wrote the manuscript. All authors read and approved the final manuscript.
